# FeIn_2_S_4_ Nanocrystals: A Ternary Metal Chalcogenide Material for Ambipolar Field‐Effect Transistors

**DOI:** 10.1002/advs.201800068

**Published:** 2018-03-27

**Authors:** Hyunjung Kim, Anand P. Tiwari, Eunhee Hwang, Yunhee Cho, Heemin Hwang, Sora Bak, Yeseul Hong, Hyoyoung Lee

**Affiliations:** ^1^ Centre for Integrated Nanostructure Physics (CINAP) Institute for Basic Science (IBS) Suwon 16419 Republic of Korea; ^2^ Sungkyunkwan University Advanced Institute of Nano Technology Sungkyunkwan University (SKKU) Suwon 16419 Republic of Korea; ^3^ Department of Chemistry Sungkyunkwan University (SKKU) Suwon 16419 Republic of Korea; ^4^ Department of Energy Science Sungkyunkwan University (SKKU) Suwon 16419 Republic of Korea

**Keywords:** ambipolar transistors, iron indium sulfide, nanocrystal field‐effect transistors

## Abstract

An ambipolar channel layer material is required to realize the potential benefits of ambipolar complementary metal–oxide–semiconductor field‐effect transistors, namely their compact and efficient nature, reduced reverse power dissipation, and possible applicability to highly integrated circuits. Here, a ternary metal chalcogenide nanocrystal material, FeIn_2_S_4_, is introduced as a solution‐processable ambipolar channel material for field‐effect transistors (FETs). The highest occupied molecular orbital and the lowest unoccupied molecular orbital of the FeIn_2_S_4_ nanocrystals are determined to be −5.2 and −3.75 eV, respectively, based upon cyclic voltammetry, X‐ray photoelectron spectroscopy, and diffraction reflectance spectroscopy analyses. An ambipolar FeIn_2_S_4_ FET is successfully fabricated with Au electrodes (*E*_F_ = −5.1 eV), showing both electron mobility (14.96 cm^2^ V^−1^ s^−1^) and hole mobility (9.15 cm^2^ V^−1^ s^−1^) in a single channel layer, with an on/off current ratio of 10^5^. This suggests that FeIn_2_S_4_ nanocrystals may be a promising alternative semiconducting material for next‐generation integrated circuit development.

## 1 Introduction

For several decades, ambipolar field‐effect transistors (FETs) have attracted considerable attention as a potential complementary metal–oxide–semiconductor (CMOS) transistor technology that could meet stringent application requirements for compactness and efficiency.^1^ Although conventional CMOS transistors provide lower power dissipation and higher density of logic functions on a chip for integrated circuits (ICs), their complicated device structure is accompanied by high cost and complex fabrication steps.^2^ Ambipolar FETs, which allow simultaneous electron and hole transport with simplified devices and fabrication processes, may be a next‐generation alternative for CMOS circuits, avoiding the need to isolate two types of unipolar transistors from each other to prevent leakage current in conventional silicon‐based devices.^3^ Therefore, there have been many efforts to demonstrate charge carrier ambipolarity in conducting channels as a means to realize the compact and simple device structure offered by ambipolar FETs.

To demonstrate ambipolar transport in a single channel layer, it is necessary for the highest occupied molecular orbital (HOMO) and the lowest unoccupied molecular orbital (LUMO) levels of the semiconducting material to be suitably positioned relative to the work functions of the electrode materials. This can be achieved by choosing materials that have appropriate bandgap structures, doping to tune the bandgap, or combining both n‐ and p‐type semiconducting materials to form the channel.^1^, ^4^ In the trend toward more efficient device manufacturing and miniaturization of device structure, there is no doubt that the intrinsic bandgap structure of materials is most essential to characterize ambipolar transport behavior. In the same context, various materials have been applied to FETs to demonstrate ambipolar transport in simple device structure. For example, combined n‐ and p‐type organic‐based FETs display ambipolar transport with great potential for cost efficiency, large‐scale production, light weight, and physical flexibility, but their low stability and mobility remain as challenges to overcome.^5^ FETs based upon carbon nanotubes, especially single‐walled nanotubes (SWNTs), have shown ambipolar transport with high field‐effect mobility (79 000 cm^2^ V^−1^ s^−1^) and a high on/off ratio (10^7^).^6^ However, the challenges of purifying and separating SWNTs selectively from multiwalled nanotubes or graphene hinder their industrial application in ambipolar transistors because these impurities are metallic in character, promoting leakage in the off state and thereby worsening the on/off ratio considerably.^7^

Additionally, inorganic nanocrystals (NCs) have emerged that exhibit electrical properties with great application potential such as wide libraries of bandgap tuning by means of doping, functionalization, and morphology and size adjustment.^8^ Moreover, because the solution process enables cost‐effective fabrication, the application of NCs as FET channel layer materials has been widely studied, especially for NCs of cadmium and lead chalcogenides.^9^ Recently, various studies were performed to improve low charge transport ability by overcoming the poor contacts arising from the large interparticle spacing between NCs^10^ using strategies such as ligand exchange, Lewis base treatment,[[qv: 10b]] or building a superlattice structure.^11^ However, the fact that only lead chalcogenides have displayed ambipolar behavior, due to their intrinsic band structure, still hinders development of NC‐based ambipolar FETs.^1^ Furthermore, there have been many efforts to achieve ambipolar transistors using lead chalcogenides that have included passivation,^12^ ligand exchange,^13^ and doping.^14^ However, the resulting devices still suffer from low mobility and poor stability under ambient conditions.[[qv: 13a]] To complicate matters further, lead is a well‐known poison and its use hinders industrial applications. Therefore, development of new NC materials demonstrating ambipolar transport is highly desirable to fully realize the promise of compact and cost‐effective IC based upon ambipolar FETs.

In the present work, we report the first demonstration of FeIn_2_S_4_ NCs as a new solution‐processable ambipolar FET channel material. We designed a device structure that allows FeIn_2_S_4_ NCs to impart ambipolar transport properties, notably by ensuring suitable band edge positions relative to the Fermi level of a noble metal electrode material, namely Au (5.1 eV). The characteristics of the band edges of the as‐synthesized FeIn_2_S_4_ NC were investigated by using X‐ray photoelectron spectroscopy (XPS), cyclic voltammetry, and diffuse reflectance spectroscopy (DRS). Our results are expected to provide both electron and hole transport ability for ambipolar FETs. The resulting electrical characteristics will be discussed with regard to *I*–*V* curves from FeIn_2_S_4_ FETs.

## 2 Results and Discussion

### 2.1 Characterizations of FeIn_2_S_4_ Nanocrystals

**Figure 1:**
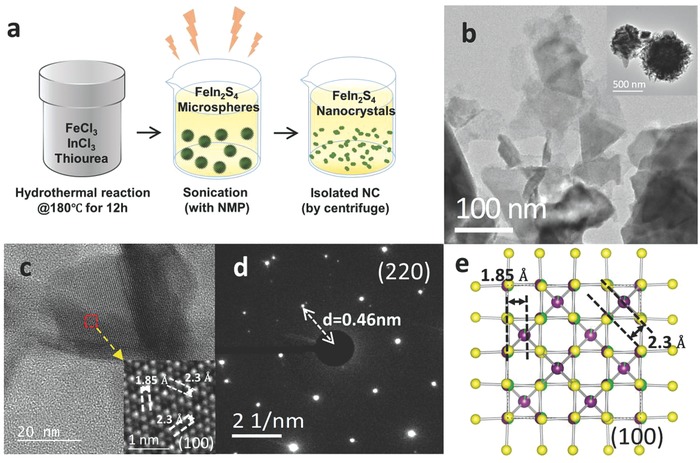
Characterization of morphology and crystallinity. a) Schematic illustration from synthesis to cleavage to obtain FeIn_2_S_4_ nanocrystals. b) TEM images of as‐synthesized flower‐like FeIn_2_S_4_ microspheres (inset) and cleaved petal‐like NCs. c) HRTEM images of FeIn_2_S_4_ NCs showing the atomic arrangement, which is preserved even after cleavage (inset). d) SAED patterns obtained from (220), showing high crystallinity after cleavage. e) Illustration of the thiospinel structure of FeIn_2_S_4_, which is identical to the inset of (c). The probability of occupation by Fe, In, and S ions is represented by green, purple, and yellow, respectively.

FeIn_2_S_4_ NCs were prepared by means of a hydrothermal reaction method described in a previous study.^15^ As‐synthesized nanoparticles were cleaved into smaller size via sonication with *N*‐methyl‐2‐pyrrolidone (NMP), such that charge transport was not hindered due to large interparticle distance but a densely packed film was formed (**Figure** **1**a and Figure S2 (Supporting Information)). To determine the crystal structure and phase of FeIn_2_S_4_ NCs, high‐resolution transmission electron microscopy (HRTEM) images were acquired (Figure 1). The as‐synthesized FeIn_2_S_4_ NCs were 500 nm in diameter (inset of Figure 1b) and then were cleaved into single‐crystalline petal‐like NCs less than 100 nm in diameter (Figure 1b). Their atomic arrangement was preserved after cleavage into smaller NCs (Figure 1c and inset), with high crystallinity that was evidenced on selected area electron diffraction (SAED) patterns (Figure 1d). The spinel structure of FeIn_2_S_4_ NC is illustrated in Figure 1e. As explained above, tetrahedral and octahedral structures occupy each FeIn_2_S_4_ unit cell, each having different probabilities of containing Fe^3+^ (green color in Figure 1e) and In^3+^ ions (purple color in Figure 1e), which is supported by the high‐resolution images obtained from the (220) plane of FeIn_2_S_4_ NCs.

**Figure 2:**
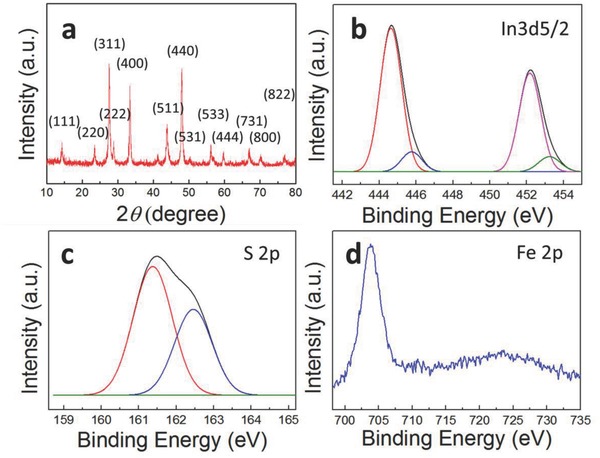
Structural and chemical characterization. a) XRD pattern of as‐synthesized FeIn_2_S_4_ NCs. b–d) XPS core level spectra of b) In 3d_5/2_, c) S 2p, and d) Fe 2p.

The crystal structure and chemical bonding of the NCs were also investigated. X‐ray diffraction (XRD) peaks can be indexed as spinel structures of FeIn_2_S_4_ (JCPDS Card No. 80‐0608 shown in Figure S1 in the Supporting Information) with peaks at 14.3°, 23.48°, 27.54°, 28.8°, 33.38°, 43.88°, 48.02°, 50.4°, 56.16°, 59.76°, 66.96°, 70.18°, and 77° corresponding to the *hkl* planes of the (111), (220), (311), (222), (400), (511), (440), (531), (533), (444), (731), (800), and (822) phases (*a* = 10.61 Å), indicating high crystallinity and no impurities (**Figure** **2**a). The chemical bonding of the compound was probed by means of XPS with the core levels of Fe 2p, In 3d, and S 2p, at respective binding energies of 702, 445, and 162 eV (Figure 2b–d). The Fe 2p binding energy peaks were of relatively low intensity (Figure 2d), suggesting relatively little bonding of iron compared with that of indium and sulfur ions. A likely explanation for this observation is that the FeIn_2_S_4_ NCs had a thiospinel structure, which can be described as AB_2_S_4_, whereas A, B, and S represent tetrahedral, octahedral structure and sulfur ions, respectively; in this unique crystal structure, the Fe^3+^ ions would occupy the octahedral sites only, whereas the In^3+^ ions would be equally distributed among the tetrahedral sites.^16^

### 2.2 Bandgap and Band Edge Investigation

**Figure 3:**
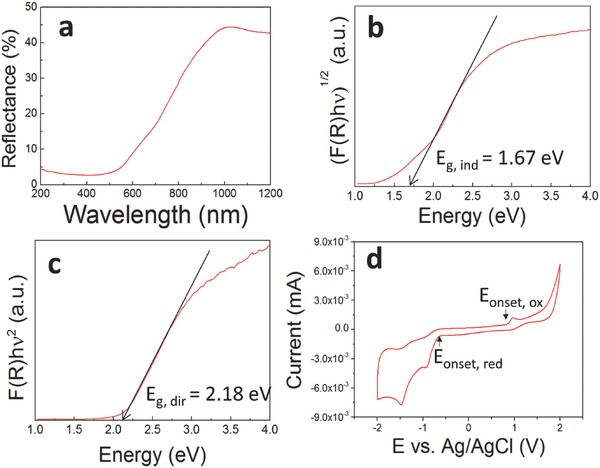
Bandgap and edge characterization of FeIn_2_S_4_ thin films. a) Diffuse reflectance spectrum of the FeIn_2_S_4_ NC film. b,c) Transformed Kubelka–Munk spectra of the FeIn_2_S_4_ NC film: b) indirect transition and c) direct transition. d) Redox voltammograms of FeIn_2_S_4_ NCs on a glassy carbon working electrode in a solvent of 0.1 m TBAPF_6_ in acetonitrile; scan rate: 200 mV s^−1^.

DRS spectra were obtained to investigate the bandgap of the FeIn_2_S_4_ NC material. From the reflectance spectrum of FeIn_2_S_4_ NCs (**Figure** **3**a), the optical absorption coefficient (α) was obtained from the Kubelka–Munk equation as

(1) $$F\left(R\right)\;=\;\alpha \;=\;{\left(1\;-\;R\right)}^{2}\mathrm{/2}R$$

where *R* is the reflectance value from the sample.^17^ To understand the optical bandgap of the FeIn_2_S_4_, the Kubelka–Munk function was used as

(2) $${\left[F\left(R\right)h\nu \right]}^{p}\;=\;A\left(h\nu \;-\;E_{g}\right)$$

where *E*_g_ is the bandgap energy, *A* is a constant representing the transition probability, and *p* is a power index that is related to the optical absorption process; *p* corresponds to allowed transitions, with a direct transition for *p* = 1/2 and an indirect transition for *p* = 2 (Figure 3b,c). Eventually, *E*_g_ can be determined by extrapolating the linear regions of each plot using *E*_g,d_ = 2.12 eV for *p* = 1/2 and *E*_g,ind_ = 1.67 eV for *p* = 2.

Because the calculated result for the optical bandgap provides only the difference between the HOMO and LUMO, the redox potential of the FeIn_2_S_4_ NC material was determined to investigate the band structure, including the conduction and valence band edges (Figure 3d). It has already been reported that the value of the optical bandgap shows good agreement with the quasiparticle gap, which can be obtained from dispersions of NCs.^18^ Furthermore, redox potentials can provide information not only regarding the *E*_g_ of NCs but also regarding the position of the HOMO and LUMO edges through calibration versus the redox coupling of ferrocene (Fc/Fc^+^) using the equations

(3) $$I_{p}\;=\;-4.8\;-\left(E_{\mathrm{ox}}\;-0.4245\right)\;\mathrm{eV}$$

and

(4) $$\mathrm{EA}\;=\;-4.8\;-\left(-E_{\mathrm{red}}\;-0.\mathrm{4245}\right)\;\mathrm{eV}$$

where *I_p_* and EA are the ionization potential and electron affinity, respectively, and the 0.4245 eV term represents the half‐wave potential of ferrocene (Figure S3, Supporting Information) relative to a reference electrode (0.01 m Ag/AgCl).^19^ A cyclic voltammogram of monodisperse FeIn_2_S_4_ NCs in a solvent of 0.1 m tetrabutylammonium hexafluorophosphate (TBAPF_6_) in acetonitrile was obtained at a scan rate of 200 mV s^−1^, which showed an *I_p_* and EA of −5.2535 and −3.7235 eV, respectively. In addition, considering the difference between *I_p_* and EA, the *E*_g_ of FeIn_2_S_4_ NCs is 1.53 eV, which is closer to the optical bandgap of the indirect transition.

**Figure 4:**
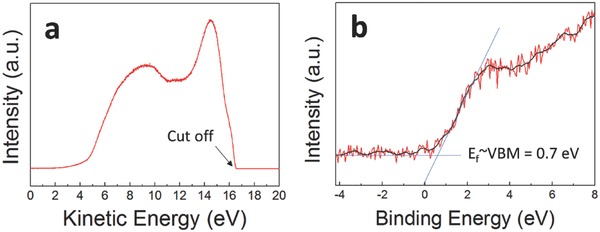
Bandgap and edge characterization of FeIn_2_S_4_ thin films. a) UPS spectrum. b) XPS valence band spectrum.

Photoelectron spectroscopy is also useful for determining the bandgap positions and Fermi level of semiconducting materials. In the present work, ultraviolet photoelectron spectroscopy (UPS) spectra were obtained to determine the absolute valence band position of FeIn_2_S_4_, and XPS spectra were acquired to determine the difference between the Fermi level and the valence band of FeIn_2_S_4_ (**Figure** **4**).^20^ Regarding band alignment in a device, to understand the charge carrier transport mechanism, it is important to establish the exact Fermi level of the semiconducting material. According to UPS spectra obtained using He I excitation (21.2 eV), the valence band position of FeIn_2_S_4_ appeared to be around −5.2 eV, with a high‐energy cutoff of 16 eV (Figure 4a). An XPS valence band maxima (VBM) spectrum showed that the difference between the *E*_F_ and VBM was around 0.7 eV (Figure 4b). As shown in XRD and XPS core level spectra of FeIn_2_S_4_ NCs, no impurities were detected that play a role of unintended dopant. It is possible to assume that *E*_F_ would be positioned in the middle of the bandgap based on the Fermi–Dirac distribution theory, which is also well matched with the results of this study.

**Figure 5:**
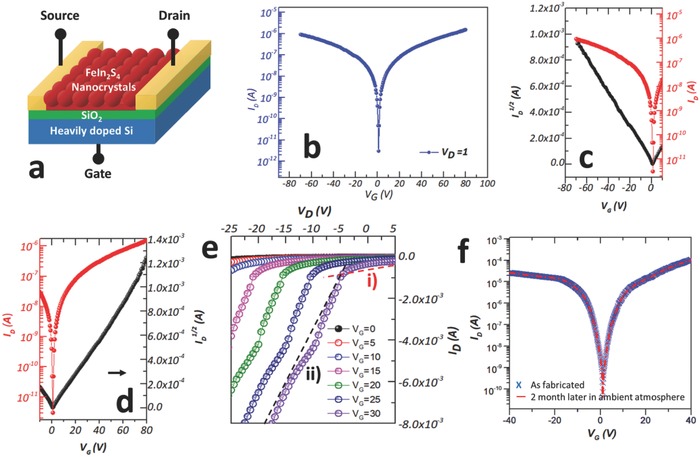
Device characteristics of FeIn_2_S_4_ NC FETs. a) Schematic illustration of FeIn_2_S_4_ FET. b) Transfer characteristics. c,d) Plots of *I*_D_ and *I*_D_^1/2^ versus *V*_G_ at constant *V*_DS_ = 1 V for ambipolar FETs assembled from FeIn_2_S_4_ NCs (*L* = 25 µm, *W* = 50 µm). e) Output characteristics: *I*_D_ versus *V*_DS_ for various *V*_G_ for a hole‐transport FET. Two different tunneling phenomena were distinguished in two voltage regions: i) direct tunneling (red dashed line) and ii) Fowler–Nordheim (FN) tunneling (black dashed line). Output characteristics for electron transport are given in Figure S5 (Supporting Information). f) Electrical characteristics were obtained for the same device after storage for two months under ambient conditions.

**Figure 6:**
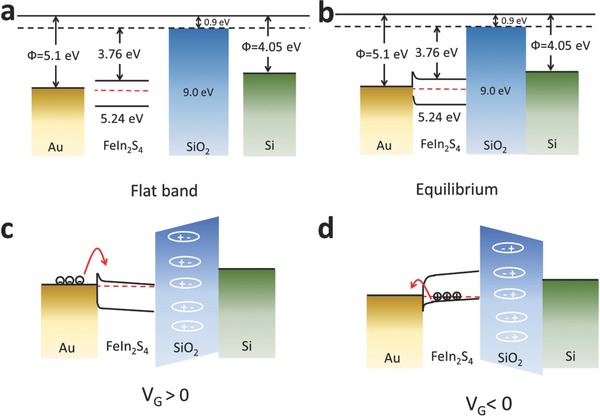
Energy band diagrams for the FeIn_2_S_4_ FET: a) flat band; b) equilibrium, *V*_G_ = 0; c) accumulation of electrons, *V*_G_ > 0; d) accumulation of holes, *V*_G_ < 0. Red arrows indicate the transfer of electrons and holes.

**Table** **1** summarizes the results of each characterization of the FeIn_2_S_4_ NCs. Since no impurities were evident in the XRD and XPS spectra, it is appropriate to assume that the Fermi level of FeIn_2_S_4_ might be located in the middle of the bandgap. From the summarized results on the band structure of the FeIn_2_S_4_ NCs (*E*_C_ = 5.25 eV and *E*_V_ = 3.72 eV), we can expect that, with most noble metal electrodes (*E*_F_ = 4.1–5.2 eV), they will show ambipolarity that can transport both electrons and holes. The ability of a transistor to operate in an ambipolar manner is determined by several factors, including the bandgaps of the semiconductor, the transport behavior of both carriers generated at the semiconductor/dielectric interface, and injection of both carriers through the interface between the metal electrodes and the channel layer.^1^ Operation as an ambipolar transistor is highly dependent on the building blocks of the device as well as the semiconductor channel layer. Therefore, the work functions of the source and drain electrodes should be considered. In the present work, 100 nm of oxidized and heavily boron‐doped Si wafer was used as the gate dielectric layer (*E*_C_ = −0.9 eV and *E*_V_ = 9.0 eV) and back gate electrode (*E*_F_ = −4.05 eV). After deposition of the channel layer by means of spin‐coating, Au source and drain electrodes (*E*_F_ = −5.1 eV) were deposited on the surface to lower the injection barriers for both charge carriers as shown in Figure S5 (Supporting Information). Finally, the transport and output current–voltage characteristics were measured. This device structure allows us to discuss both charge carrier transport mechanisms that take place at the interface. Further details will be provided with **Figures** **5** and **6**.

**Table 1:** Band energy characteristics of FeIn_2_S_4_ NCs. The band energy characteristics are measured by means of diffuse reflectance spectroscopy, electrochemical measurements, and photoelectron spectroscopy. VBM, *E*_F_, CBM, and *E*_g_ represent the valence band maximum, Fermi level, conduction band maximum, and bandgap versus vacuum level, respectively

Method	VBM [eV]	*E*_F_ [eV]	CBM [eV]	Δ*E*_g_
Electrochemical measurement	−5.25		−3.72	1.53 eV
Photoelectron spectroscopy	−5.2	−4.5		
Diffuse reflectance spectrum				1.67 eV (indirect), 2.12 eV (direct)

### 2.3 Electrical Characteristics of FeIn_2_S_4_ FETs

The structure of FeIn_2_S_4_ FET device is illustrated schematically in Figure 5a. Transfer (drain current vs gate voltage, *I*_D_ vs *V*_G_) and output (*I*_D_ vs drain voltage *V*_D_) characteristics were measured at room temperature (Figure 5b–e). V‐shape transfer curves representing typical ambipolar transfer characteristics were observed. Both the electron and hole mobilities in the linear and saturation regimes were extracted using the equation

(5) $$\mu_{\mathrm{lin}}\;=\;\frac{L}{WC_{i}\left(\frac{dI_{D}}{dV_{G}}\right)}$$

(6) $$\mu_{\mathrm{sat}}\;=\;\frac{2L}{W}C_{i}\left(\frac{d\sqrt{I_{\mathrm{DS}}}}{dV_{G}}\right)$$

where *W* and *L* are the channel width and length, and *C*_i_ is the capacitance of the 100 nm SiO_2_ (= 3.45 × 10^−8^ F cm^−2^).[[qv: 10a]] From the transfer curve, the electron and hole mobilities (*V*_DS_ = 1 V) in the linear regime were 14.96 and 9.15 cm^2^ V^−1^ s^−1^, and the mobilities (*V*_DS_ = 1 V) in the saturation regime were 0.27 and 0.2 cm^2^ V^−1^ s^−1^, respectively. The on/off current ratios were found to be 10^5^ for both electron and hole conduction. From these results, it is clear that the Fermi level of the Au was aligned close to the center of the gap between the HOMO and LUMO levels of FeIn_2_S_4_ NCs, due to Fermi level pinning phenomenon. A high Schottky barrier junction current was observed (Figure 5e), which is frequently observed in ambipolar transistors.^21^ The high mobility obtained in Figure 5c,d can be explained by a surfactant effect. In the fabrication procedure to obtain the FeIn_2_S_4_ NCs, no organic surfactant was used as shown in XPS (Figure 2b–d). The absence of a passivated organic surfactant that has different energy levels and low conductivity^2^ enhanced the transfer of charge carriers through the interparticles.

To help explain the transport behavior between the interfaces of each of the building blocks during device operation, Figure 6 gives the schematic band alignment for each of the channel layers, namely the FeIn_2_S_4_ NC film, the SiO_2_ gate dielectric, the heavily boron‐doped Si gate electrode, and the Au electrode. Figure 6a shows the flat band state, and Figure 6b shows the corresponding energy band diagram under equilibrium conditions. Under positive gate voltage, the electrons increase in concentration because they accumulate at the interface between the gate dielectric and the channel layer, which is accompanied by a shift in the bandgap to a higher potential energy (Figure 6c). Eventually, as the conduction band of the channel layer approaches the Fermi level of the source and drain electrodes, electrons can tunnel through the interface of the junction. Contrastingly, under a negative back gate voltage, because holes can accumulate at the interface between the channel layer and the gate dielectric, holes can travel through the interface and thus act as major carriers (Figure 6d). The electron mobility appears slightly higher than the hole mobility in the transfer curves because the middle of the bandgap of FeIn_2_S_4_ is shifted slightly higher than the Fermi level of the Au electrodes. This result agrees with the bandgap characterizations obtained from cyclic voltammetry and photoelectron spectroscopy measurements.

In heterojunctions, especially metal–semiconductor junctions, triangular Schottky barriers inevitably form at the interface as a result of Fermi level pinning.^22^ According to the Schottky–Mott rule, the height of the Schottky barrier is determined by the difference in energy states between the work function of the metal and the conduction band of the semiconductor, as expressed by the equation

(7) $$\Phi_{\mathrm{Bn}}\;=\;\Phi_{m}\;-\;X$$

where *Φ*_Bn_ is the Schottky barrier height, *Φ*_m_ is the work function of the metal, and *X* is the electron affinity of the semiconductor.^23^ Based on this, the Schottky barrier height between FeIn_2_S_4_ and Au is around 0.6–0.8 eV, which is not negligible for charge carrier transport. Through this barrier, carriers can tunnel through the interface by means of two phenomena: direct tunneling and Fowler–Nordheim (FN) tunneling.^24^ Two significantly different slopes are observed in the curve of current versus drain voltage in two different voltage regimes (Figure 5e(i),(ii) and Figure S6 (Supporting Information)), so it can be assumed that there is a Schottky barrier at the interface between the channel layer and the electrodes, in good agreement with the characterization results for FeIn_2_S_4_ NCs (Table 1).

Furthermore, the fabricated FET device showed high stability under ambient conditions for two months (Figure 5f) and also exhibited superior mobility and on/off ratio to lead chalcogenide‐based FETs. Thus, FeIn_2_S_4_ provides not only an efficient and simplified fabrication process owing to the solution processable and electrically stable characteristics, but also yields compact logic circuits resulting via its ambipolar transport ability, high mobility, and superior on/off ratio. Therefore, FeIn_2_S_4_ ternary metal chalcogenide NCs show considerable promise for use in next‐generation electronic devices.

## 3 Conclusion

A transition metal chalcogenide material of FeIn_2_S_4_ NCs was synthesized, and various characterizations including DRS, cyclic voltammetry, XPS, and UPS were performed to demonstrate its feasibility for use as an ambipolar material with an appropriate bandgap and band edge levels for application in ambipolar FETs. The FeIn_2_S_4_ NC FET device fabricated by means of solution processing showed ambipolar properties, high electron and hole mobilities, high on/off current ratio, and good stability even under ambient conditions. Owing to its cost‐effective fabrication and ability to be used in ambipolar FETs for compact CMOS circuits, the FeIn_2_S_4_ NC material reported herein appears promising as a next‐generation semiconducting material.

## 4 Experimental Section

*Materials*: A previously reported procedure was followed to synthesize FeIn_2_S_4_ microspheres.^15^ Because the size and morphology of the resulting materials was not yet suitable for use in the FET channel layer, the microspheres were cleaved and isolated by means of lengthy sonication in the solution phase (Figure S2, Supporting Information). Because nanoscale particles are needed to ensure sufficient charge transfer between particles during device operation, the FeIn_2_S_4_ particles were purified to collect those of sufficiently small size. The reagents used to carry out the hydrothermal reaction of FeIn_2_S_4_, namely iron chloride (FeCl_3_), indium chloride (InCl_3_), and thiourea, were purchased from Sigma‐Aldrich. All reactants were used as received. Into 40 mL of deionized (DI) water, 1 mmol of FeCl_3_, 2 mmol of InCl_3_, and 4 mmol of thiourea were dissolved, and the resulting solution was stirred at room temperature for 1 h under N_2_ gas bubbling in a two‐neck round‐bottom flask. After stirring, the solution was transferred into a Teflon‐lined stainless steel autoclave and heated for 12 h at 180 °C to carry out the reaction. The reaction solution was then cooled to room temperature and filtered. The product thus obtained was washed with DI water and then with ethanol, and then dried under vacuum at 40 °C prior to measurement.

*FET Device Fabrication*: SiO_2_ gate dielectrics (100 nm) thermally grown on heavily boron‐doped Si substrates were used; these were subjected to piranha treatment prior to use. The FeIn_2_S_4_ NCs were dispersed in NMP at 10 mg mL^−1^ concentration and then sonicated for 10 h. After sonication, there were no precipitated particles. The substrate was washed with DI water several times and then the FeIn_2_S_4_ NC solution was spin‐coated onto the substrate at 4000 rpm for 3 min; the coated substrate was soft annealed at 150 °C for 3 min to remove residual solvent. Before depositing the source and drain electrodes, the NC‐coated substrates were thermally annealed at 300 °C for 1 h. Finally, Ti/Au (5 nm/45 nm) electrodes were evaporated over the annealed coating to form band‐aligned contacts for ambipolar transport.

*Characterization and Measurements*: To characterize the FeIn_2_S_4_ NCs, XRD, XPS, UPS, and DRS analyses were performed. All Si substrates used were first cleaned with piranha solution, rinsed with DI water several times, blown dry with N_2_, and then treated with plasma under an oxygen atmosphere. To prepare NC samples for analysis, FeIn_2_S_4_ NC solution (50 mg mL^−1^) was drop cast onto substrates and dried under vacuum at 70 °C overnight. To investigate the crystallinity and crystal structure of the FeIn_2_S_4_ NCs, the prepared samples were characterized via XRD (Rigaku Ultima IV) and transmission electron microscopy (TEM; JEOL JEM‐2100F). To obtain information on the chemical binding and bandgap of FeIn_2_S_4_ NCs, XPS measurements were performed using a Thermo VG Microtech ESCA 2000 equipped with a monochromatic Al‐Kα X‐ray source and operated at 100 W. Cyclic voltammograms were obtained using a CHI660c electrochemical workstation. To investigate the optical energy bandgap of the FeIn_2_S_4_ NCs, DRS was conducted using a UV‐3600 (Shimadzu Corp.); BaSO_4_ was used as the standard. Data on source–drain current versus back‐gate voltage for the FET devices were collected using a 4200 Keithley semiconductor characterization system operated at room temperature in a vacuum within the range of 1 × 10^−4^–1 × 10^−5^ Torr.

## Conflict of Interest

The authors declare no conflict of interest.

## Supporting information

Supplementary
