# Supplementary material for: FeIn2S4 Nanocrystals: A Ternary Metal Chalcogenide Material for Ambipolar Field‐Effect Transistors
Source: Adv Sci (Weinh). 2018 Mar 27;5(7):1800068. doi: 10.1002/advs.201800068 (PMC6051185; doi:10.1002/advs.201800068)
Supplement: Supplementary file 1 — Supplementary [file ADVS-5-1800068-s001.pdf]

Copyright WILEY-VCH Verlag GmbH & Co. KGaA, 69469 Weinheim, Germany, 2018.

Supporting Information

for *Adv. Sci.,* DOI: 10.1002/advs.201800068

$FeIn_2S_4$ Nanocrystals: A Ternary Metal Chalcogenide Material for Ambipolar Field-Effect Transistors

*Hyunjung Kim, Anand P. Tiwari, Eunhee Hwang, Yunhee Cho, Heemin Hwang, Sora Bak, Yeseul Hong, and Hyoyoung Lee**

Copyright WILEY-VCH Verlag GmbH & Co. KGaA, 69469 Weinheim, Germany, 2016.

Supporting Information

FeIn2S4 Nanocrystals: A Ternary Metal Chalcogenide Material for Ambipolar Field Effect Transistors

*Hyunjung Kim[1,2], Anand P. Tiwari[2,3], Eunhee Hwang[2,3], Yunhee Cho[2,3], Heemin Hwang[2,4], Sora Bak[2,3], Yeseul Hong[3] and Hyoyoung Lee[1,2,3,4]**

In this supplement, we provide the following data and description which support to our main text:

Figure S1: XRD

Figure S2: Images of as-synthesized FeIn2S4 nanocrystals dispersed in diverse solvent

Figure S3: Redox potential of Ferrocene

Figure S4: Cross-sectional SEM images of $FeIn_2S_4$ FETs

Figure S5: Output characteristics for electron transport in $FeIn_2S_4$ FETs

Figure S6. The SEM image obtained from $FeIn_2S_4$ ambipolar FETs in top view before deposition of electrodes.

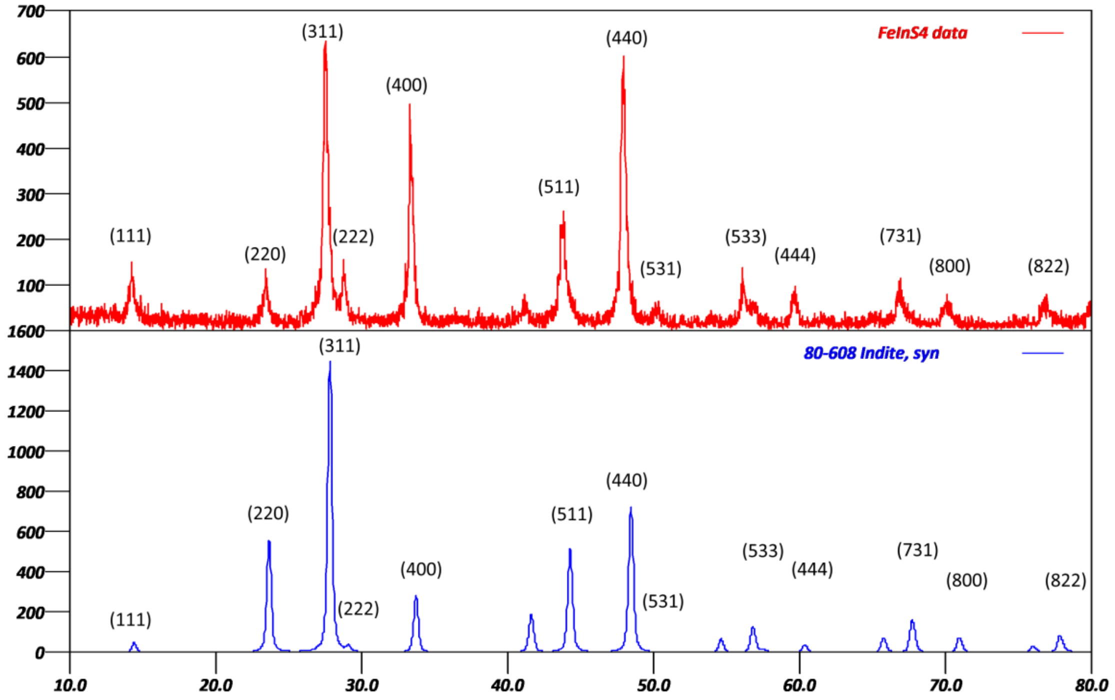

Figure S1. XRD spectrum of as synthesized $FeIn_2S_4$ nanocrystals and the reference JCPDS Card No. 80-0608, a = 10. 61Å is shown without impurities with high crystallinity.

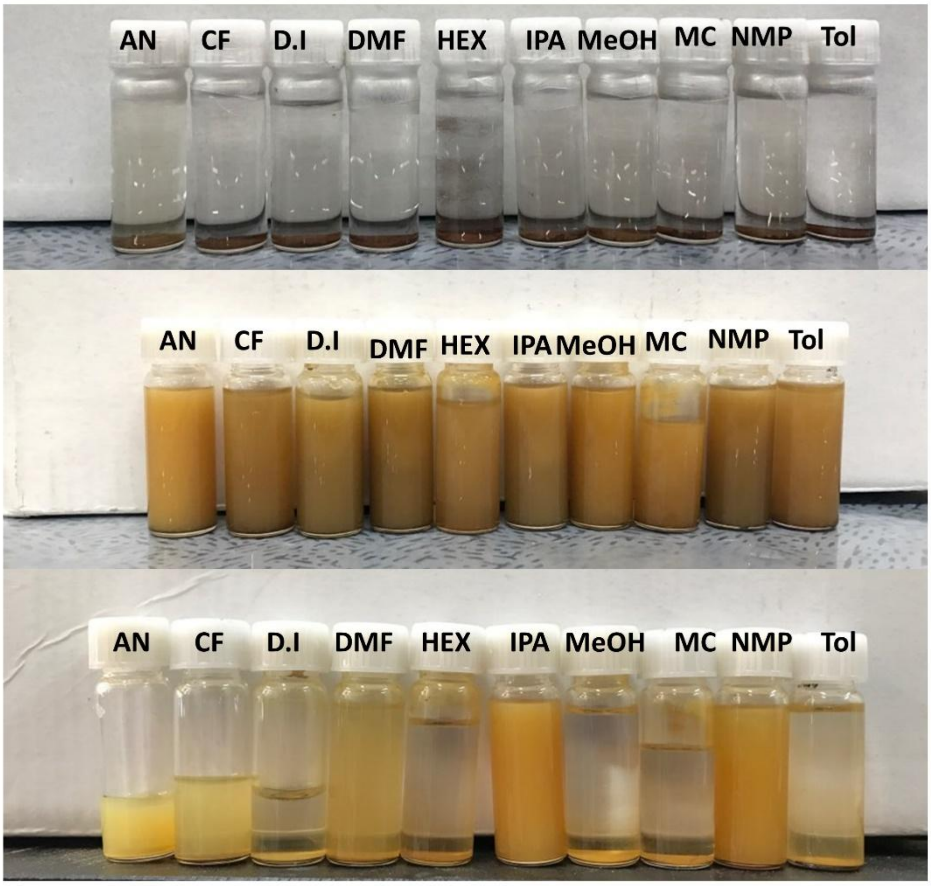

Figure S2. The solutions obtained via sonication for more than a day in a different solvent. From the result of as-dispersed, (the top image), as-sonicated (the center image), and 1 hour stored solutions (the bottom image) showing different dispersability. NMP and IPA show fine particle distribution and dispersability.

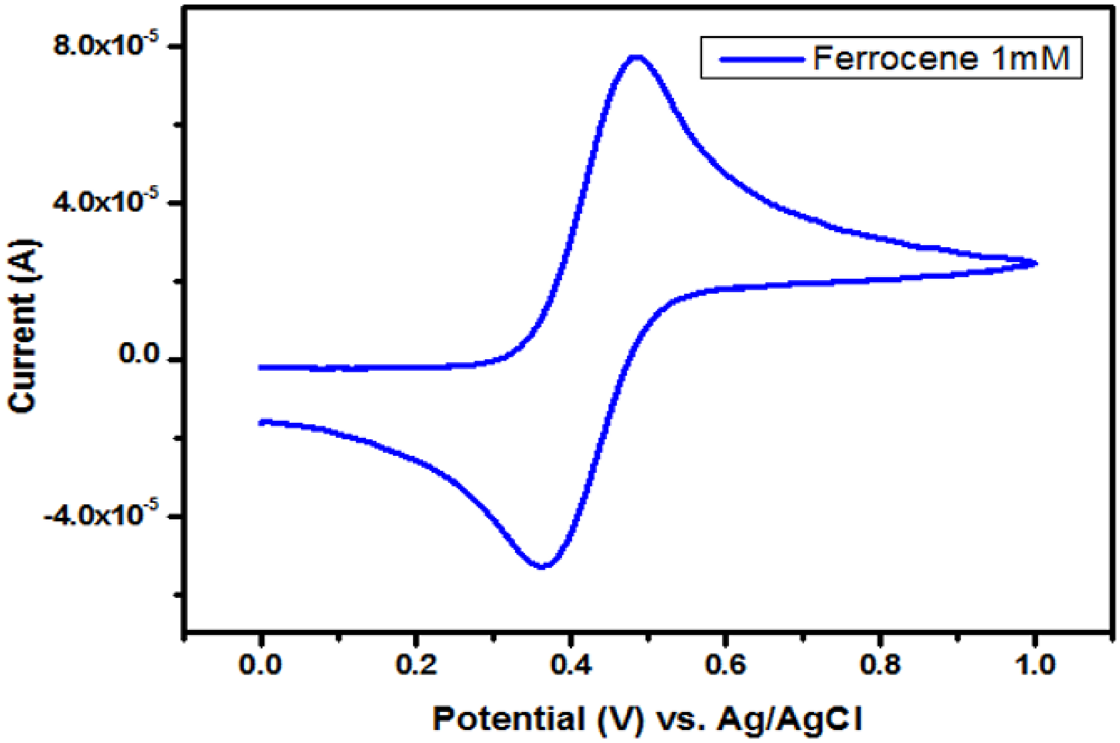

Figure S3. Redox potential of ferrocene 1 mN in 0.1 M TABPF acetonitrile solution, which was used for the calculation of HOMO and LUMO level in cyclic voltammetry using the equation for $E_{1/2}$ (ferrocene) = half-wave potential of ferrocene = ½(0.484+0.365) = 0.4245 and HOMO (or LUMO) (eV) = -4.8-(Eonset-$E_{1/2}$(ferrocene)).

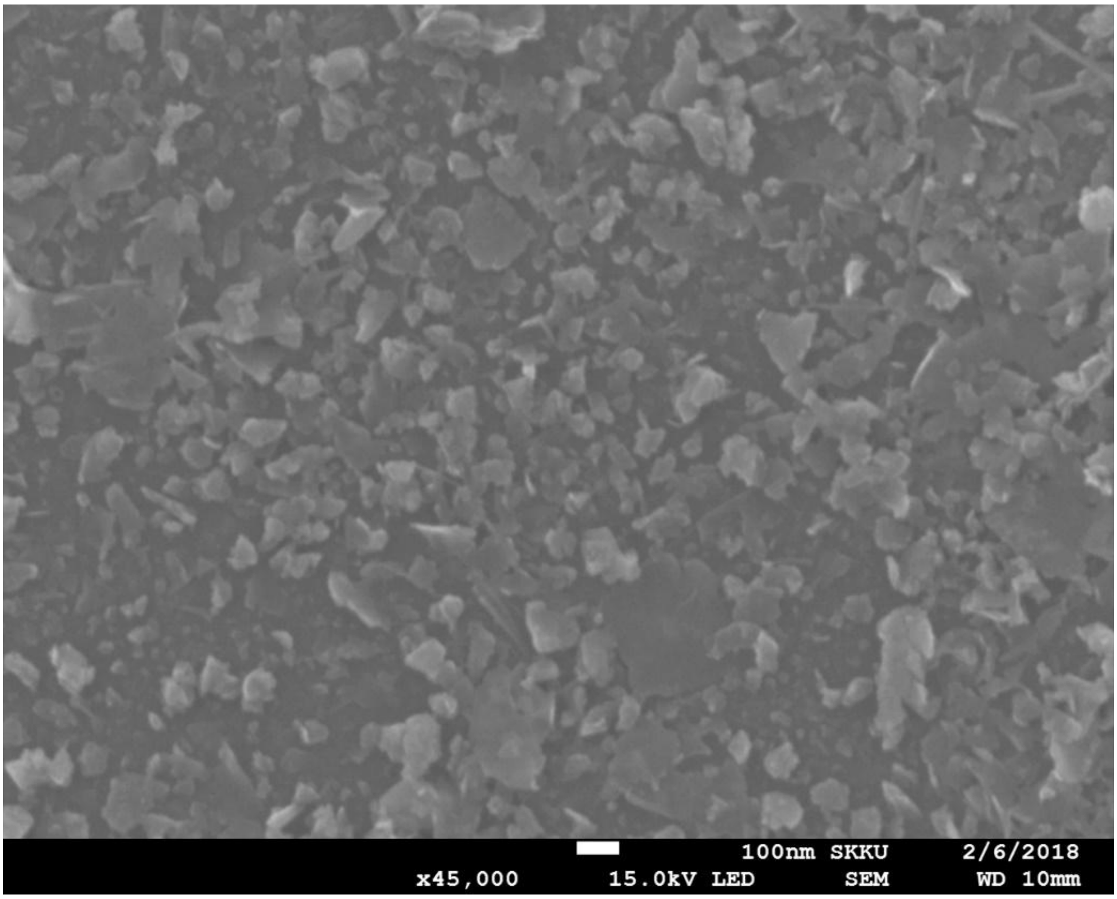

Figure S4. The SEM image obtained from $FeIn_2S_4$ ambipolar FETs (top view) before deposition of electrodes.

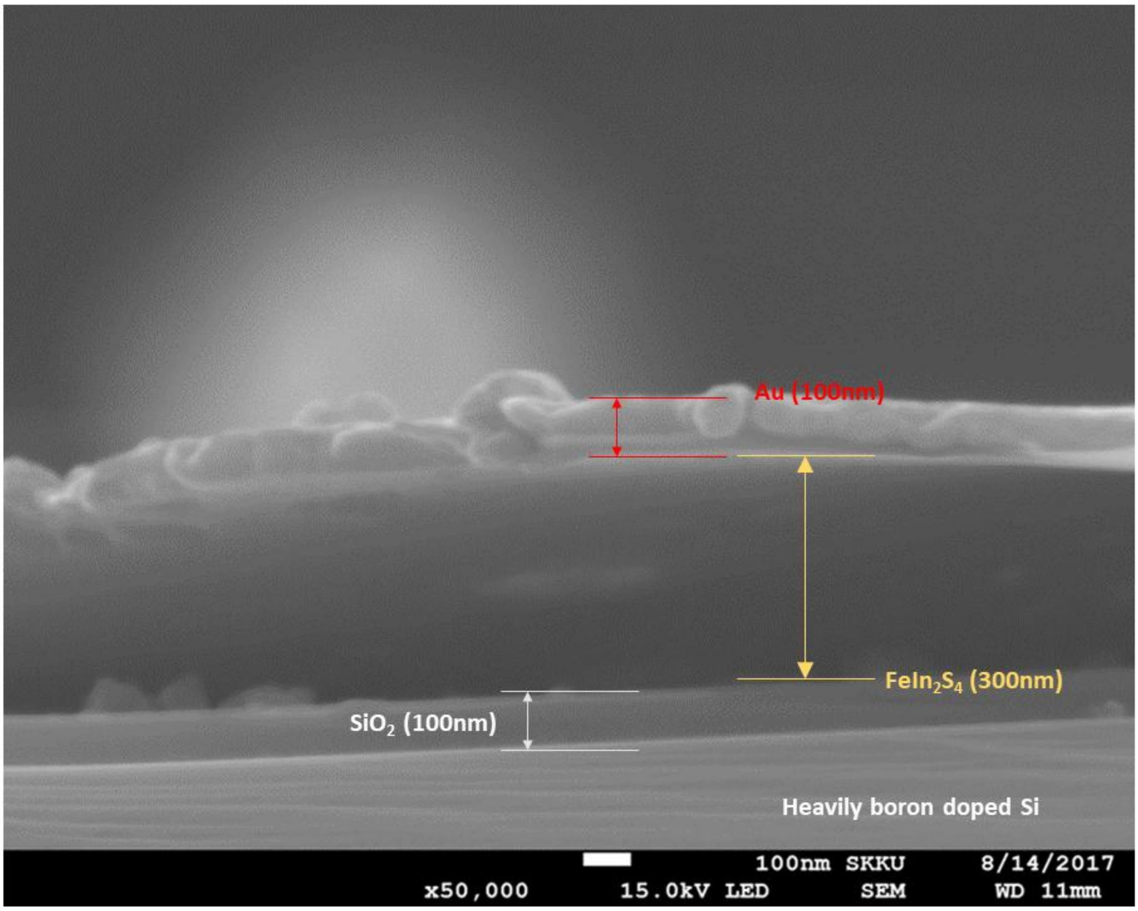

Figure S5. The SEM image obtained from $FeIn_2S_4$ ambipolar FETs (cross-sectional view).

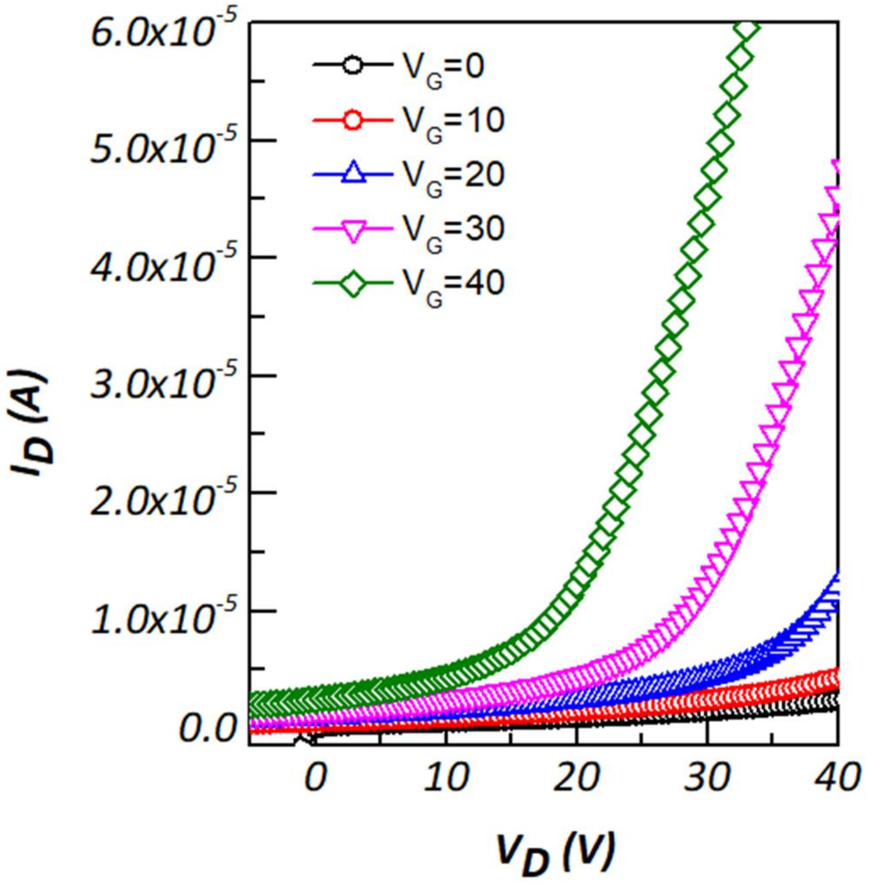

Figure S6. Output characteristics with the plot of I_D versus V_{DS} as a function of V_G for electron transport FETs.

[1] S. Z. Bisri, C. Piliego, J. Gao, M. A. Loi, *Advanced Materials* **2014**, *26*, 1176-1199.

[2] aE. C. P. Smits, T. D. Anthopoulos, S. Setayesh, E. van Veenendaal, R. Coehoorn, P. W. M. Blom, B. de Boer, D. M. de Leeuw, *Physical Review B* **2006**, *73*, 205316; bE. Fortunato, P. Barquinha, R. Martins, *Advanced Materials* **2012**, *24*, 2945-2986.

[3] Y. F. Lin, Y. Xu, S. T. Wang, S. L. Li, M. Yamamoto, A. Aparecido-Ferreira, W. Li, H. Sun, S. Nakaharai, W. B. Jian, K. Ueno, K. Tsukagoshi, *Advanced Materials* **2014**, *26*, 3263-3269.

[4] C. Zhou, Y. Zhao, S. Raju, Y. Wang, Z. Lin, M. Chan, Y. Chai, *Advanced Functional Materials* **2016**, *26*, 4223-4230.

[5] M. Muccini, *Nat Mater* **2006**, *5*, 605-613.

[6] aT. Dürkop, S. A. Getty, E. Cobas, M. S. Fuhrer, *Nano Letters* **2004**, *4*, 35-39; bL. Yu-Ming, J. Appenzeller, J. Knoch, P. Avouris, *IEEE Transactions on Nanotechnology* **2005**, *4*, 481-489.

[7] F. Hennrich, W. Li, R. Fischer, S. Lebedkin, R. Krupke, M. M. Kappes, *ACS Nano* **2016**, *10*, 1888-1895.

[8] aM. V. Kovalenko, L. Manna, A. Cabot, Z. Hens, D. V. Talapin, C. R. Kagan, V. I. Klimov, A. L. Rogach, P. Reiss, D. J. Milliron, American Chemical Society, **2015**; bM.

A. Boles, D. Ling, T. Hyeon, D. V. Talapin, *Nature materials* **2016**, *15*, 141-153; cA. Sahu, M. S. Kang, A. Kompch, C. Notthoff, A. W. Wills, D. Deng, M. Winterer, C. D. Frisbie, D. J. Norris, *Nano letters* **2012**, *12*, 2587-2594.
[9] D. V. Talapin, J.-S. Lee, M. V. Kovalenko, E. V. Shevchenko, *Chemical reviews* **2010**, *110*, 389.
[10] aJ. Jang, D. S. Dolzhnikov, W. Liu, S. Nam, M. Shim, D. V. Talapin, *Nano letters* **2015**, *15*, 6309-6317; bD. V. Talapin, C. B. Murray, *Science* **2005**, *310*, 86-89.
[11] Y. Liu, M. Gibbs, J. Puthussery, S. Gaik, R. Ihly, H. W. Hillhouse, M. Law, *Nano Letters* **2010**, *10*, 1960-1969.
[12] M. I. Nugraha, R. Häusermann, S. Z. Bisri, H. Matsui, M. Sytnyk, W. Heiss, J. Takeya, M. A. Loi, *Advanced Materials* **2015**, *27*, 2107-2112.
[13] aY. Liu, J. Tolentino, M. Gibbs, R. Ihly, C. L. Perkins, Y. Liu, N. Crawford, J. C. Hemminger, M. Law, *Nano letters* **2013**, *13*, 1578-1587; bS. Z. Bisri, C. Piliego, M. Yarema, W. Heiss, M. A. Loi, *Advanced Materials* **2013**, *25*, 4309-4314; cW.-k. Koh, S. R. Saudari, A. T. Fafarman, C. R. Kagan, C. B. Murray, *Nano letters* **2011**, *11*, 4764-4767; dS. J. Oh, Z. Wang, N. E. Berry, J.-H. Choi, T. Zhao, E. A. Gaulding, T. Paik, Y. Lai, C. B. Murray, C. R. Kagan, *Nano letters* **2014**, *14*, 6210-6216.
[14] M. S. Kang, A. Sahu, C. D. Frisbie, D. J. Norris, *Advanced Materials* **2013**, *25*, 725-731.
[15] X. Chen, Z. Zhang, X. Zhang, J. Liu, Y. Qian, *Journal of Crystal Growth* **2005**, *277*, 524-528.
[16] aR. J. Hill, J. R. Craig, G. V. Gibbs, *Journal of Physics and Chemistry of Solids* **1978**, *39*, 1105-1111; bQ. Lu, J. Hu, K. Tang, Y. Qian, G. Zhou, X. Liu, *Chemistry letters* **1999**, *28*, 481-482.
[17] H.-S. Kim, C.-R. Lee, J.-H. Im, K.-B. Lee, T. Moehl, A. Marchioro, S.-J. Moon, R. Humphry-Baker, J.-H. Yum, J. E. Moser, M. Grätzel, N.-G. Park, *Scientific Reports* **2012**, *2*, 591.
[18] aS. N. Inamdar, P. P. Ingole, S. K. Haram, *ChemPhysChem* **2008**, *9*, 2574-2579; bS. K. Poznyak, N. P. Osipovich, A. Shavel, D. V. Talapin, M. Gao, A. Eychmüller, N. Gaponik, *The Journal of Physical Chemistry B* **2005**, *109*, 1094-1100.
[19] E. Kucur, J. Riegler, G. A. Urban, T. Nann, *The Journal of chemical physics* **2003**, *119*, 2333-2337.
[20] aW.-J. Chun, A. Ishikawa, H. Fujisawa, T. Takata, J. N. Kondo, M. Hara, M. Kawai, Y. Matsumoto, K. Domen, *The Journal of Physical Chemistry B* **2003**, *107*, 1798-1803; bL. Ley, R. Pollak, F. McFeely, S. P. Kowalczyk, D. Shirley, *Physical Review B* **1974**, *9*, 600; cE. P. Nguyen, B. J. Carey, J. Z. Ou, J. van Embden, E. D. Gaspera, A. F. Chrimes, M. J. Spencer, S. Zhuiykov, K. Kalantar-zadeh, T. Daeneke, *Advanced Materials* **2015**, *27*, 6225-6229.
[21] S. Das, M. Demarteau, A. Roelofs, *ACS nano* **2014**, *8*, 11730-11738.
[22] F. Léonard, J. Tersoff, *Physical Review Letters* **2000**, *84*, 4693-4696.
[23] C. Kim, I. Moon, D. Lee, M. S. Choi, F. Ahmed, S. Nam, Y. Cho, H.-J. Shin, S. Park, W. J. Yoo, *ACS nano* **2017**, *11*, 1588-1596.
[24] M.-H. Doan, Y. Jin, S. Adhikari, S. Lee, J. Zhao, S. C. Lim, Y. H. Lee, *ACS Nano* **2017**, *11*, 3832-3840.
